# Response to immunotherapy plus anti‐angiogenic agent combination challenges hepatocellular carcinoma prognostic scores

**DOI:** 10.1002/hep4.2047

**Published:** 2022-07-12

**Authors:** Xavier Adhoute, Thomas Wolf

**Affiliations:** ^1^ Department of Gastroenterology and Hepatology Hôpital Saint‐Joseph Marseille France

## Abstract

This new UHPI score identifies three groups with different prognosis, but with no clear therapeutic guidance. Moreover, this model's predictive ability is now challenged in the era of combination therapies where durable responses are more commonly observed, and where response criteria remain to be defined.

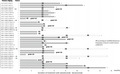

To the editor,

We read with interest the study by Demirtas et al.^[^ ^1^ ^]^ who proposed a new based‐point score, named the unresectable hepatocellular carcinoma (HCC) prognostic index (UHPI), assessing the risk of death at 6 months and 1 year for nonsurgical HCCs. Based on an HCC population classified as Barcelona Clinic Liver Cancer (BCLC) stage B, C, and D and mostly treated with transarterial chemoembolization, this index identifies three different prognostic groups and outperforms other classifications (BCLC system) or scores (in particular the nodularity, infiltrative, alpha‐fetoprotein, Child‐Pugh stage, Eastern Cooperative Oncology Group performance [NIACE] score that we proposed a few years ago). While the results are evident, they deserve several comments.

First, the prognostic performance of a new score is commonly better than that of other existing systems because it is driven by prior studies. Indeed, the UHPI model pools patients classified as prognostic score (PS) 0 or 1, as previously suggested to improve the BCLC system prognostic value.^[^ ^2^ ^]^

Second, this new model includes well‐known prognostic parameters in this setting (e.g., tumor and patient characteristics, liver function). However, the threshold values and coefficients (0.5/1/2) are highly related to the training cohort. Therefore, the UHPI model reproducibility may be compromised along with its predictive ability for patients near the cut‐off values. Point‐based scores are less powerful than continuous variable models.

Third, a model should be able to help in therapeutic decisions. Regarding unresectable HCC, there is a clear consensus on PS and liver function as essential criteria for a therapeutic decision as they may constitute real contraindications. Conversely, tumor characteristics, as pejorative as they may be, are in the era of combination therapy with immune checkpoint inhibitors (directed against programmed death receptor‐1 ligand) plus antibodies targeting vascular endothelial growth factor A less decisive because they do not prevent a durable tumor response as predictive criteria for a response are not yet known. Our unit treated 35 consecutive patients with unresectable HCC (classified according to BCLC, NIACE, and UHPI systems) from August 2020 with the atezolizumab–bevacizumab combination (Figure 1).^[^ ^3^ ^]^ Despite a high UHPI or NIACE score, durable responses were observed. Thus, the treatment overcame the tumor parameters and changed the prognosis. We did not experience this in the tyrosine kinase inhibitor era.

**FIGURE 1:**
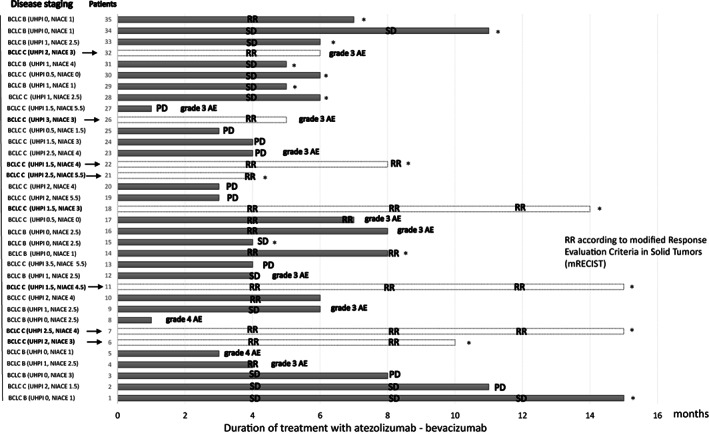
Patients with unresectable hepatocellular carcinoma being treated with atezolizumab–bevacizumab (*n* = 35). *indicates patients with ongoing treatment; the arrow indicates patients with radiologic response and higher scores; RR is according to the modified Response Evaluation Criteria in Solid Tumors. AE, adverse event; BCLC, Barcelona Clinic Liver Cancer; NIACE, nodularity, infiltrative, alpha‐fetoprotein, Child‐Pugh stage, Eastern Cooperative Oncology Group performance; PD, progressive disease; RR, radiologic response; SD, stable disease; UHPI, unresectable hepatocellular carcinoma prognostic index.

In conclusion, this new UHPI score identifies three groups with different prognoses but with no clear therapeutic guidance. Moreover, this model's predictive ability is now challenged in the era of combination therapies where durable responses are more commonly observed and where response criteria remain to be defined (e.g., oncogenic alterations, immune markers).

## AUTHOR CONTRIBUTIONS

Xavier Adhoute and Thomas Wolf are physicians in charge of the patients, and both wrote the letter.

## CONFLICTS OF INTEREST

Xavier Adhoute consults for Bayer and Servier; he consults for and received grants from Ipsen, Mylan Medical, and Gilead. Thomas Wolf has nothing to report.
